# Inclination of mandibular incisors and symphysis in severe skeletal class III malocclusion

**DOI:** 10.1186/s13005-023-00361-6

**Published:** 2023-05-10

**Authors:** Jieni Zhang, Yuqi Liang, Rui Chen, Si Chen, Jiuxiang Lin, Bing Han, Xiaomo Liu

**Affiliations:** 1 Department of Orthodontics, Peking University School and Hospital of Stomatology & National Center for Stomatology & National Clinical Research Center for Oral Diseases & National Engineering Laboratory for Digital and Material Technology of Stomatology & Beijing Key Laboratory of Digital Stomatology & Research Center of Engineering and Technology for Computerized Dentistry Ministry of Health & NMPA Key Laboratory for Dental Materials, 22 Zhongguancun South Avenue, Haidian District 100081 Beijing, China; 2 Yuncheng Stomatological Hospital, Yuncheng, China

**Keywords:** Skeletal class III malocclusion, Lower incisor, Cephalometry

## Abstract

**Objective:**

The aim of this study was to systematically explore the inclination of the lower central incisor and symphysis in alveolar bone in severe skeletal class III patients.

**Materials and methods:**

A total of 198 severe skeletal class III patients (ANB ≤ -4°) who underwent combined orthodontic and orthognathic treatment were divided into three groups based on the mandibular plane angle (MP-SN). Pretreatment lateral cephalograms were analysed and compared among the three groups. We also assessed cone-beam computed tomography (CBCT) images of 11 samples to investigate the reliability of the cephalometric analysis.

**Results:**

ANOVA showed no statistically significant differences in the angle between the long axis of the mandibular symphysis and the long axis of the lower central incisor (MIA) among the low-angle, normal-angle and high-angle groups (*P* > 0.05), while significant differences were found in the angle between the axis of the lower incisor and the mandibular plane (IMPA) among the three groups (*P* < 0.001). The mean IMPA decreased with increasing MP-SN in the 198 patients. The mean MIA in the low-angle and normal-angle groups was 3.70° and 3.52°, respectively, while the value (2.33°) was smaller in the high-angle group. Paired-samples t test showed no statistically significant differences between the cephalometric and CBCT measurements of the MP-SN, the angle between the mandibular plane and the Frankfort plane (FH-MP) and the MIA (*P* > 0.05).

**Conclusions:**

In severe skeletal class III patients, the long axis of the lower central incisor was highly consistent with the long axis of the mandibular symphysis, which was more obvious in the high-angle subjects. The MIA reflects the physiological inclination of the lower central incisor better than the IMPA.

## Introduction

Class III malocclusion is a common orthodontic malocclusion that often manifests not only in the dental arches but also as skeletal discrepancy [1, 2]. Patients with class III malocclusion often seek orthodontic treatment due to an anterior crossbite and concave profile. In severe skeletal class III patients, the lower incisors are more lingually inclined for compensation. Studies have shown that the associated alveolar bone around lower incisors is also more lingually inclined and is originally or developmentally thinner than normal occlusion [3–5]. Currently, with the development of orthodontics and societal progress, the demand for healthy orthodontics has increased [6, 7]. Professor Lin Jiuxiang at Peking University proposed the concept of healthy orthodontics while pursuing facial and dental improvements in 2018. The concept emphasizes that efficient tooth movement and alveolar bone reformation effects could be realized under light force. In addition, teeth should be maintained within the base bone during treatment, thus avoiding the risk of fenestration and dehiscence [8].

Previous studies on the inclination of the lower incisors mainly measured the angle between the axis of the lower incisor and the mandibular plane (IMPA) [9, 10]. Presurgical orthodontics in severe skeletal class III patients removes the dental compensation for the jaw deformity, thereby retracting the maxillary incisors and proclining the lower incisors to facilitate surgical movements. However, complete decompensation based on the index IMPA might cause the incisors to exceed the alveolar bone house, leading to fenestration, dehiscence and other unacceptable side effects [4, 11]. Therefore, a more suitable indicator is needed to evaluate the inclination of the mandibular incisors.

Previously, our research group was the first to propose the concept of the MIA, namely, the angle between the long axis of the mandibular symphysis (MA) and the long axis of the lower central incisor (IA), which are used to describe the orientation of the mandibular symphysis and the inclination of the lower incisors. Two studies proposed the MIA as a new index to reflect the inclination of the lower central incisor and investigated the consistency between the IA and MA in natural dentition and in patients treated by the force-transmission technique [12, 13]. The MIA refers to the angle formed between the IA and MA. MA is the line from the midpoint of the line of the labial and lingual lower alveolar edge and the centre point of the mandibular symphysis.

We paid more attention to the lower incisor maintained in the alveolar base bone during treatment. The more direct measurement index reflecting the relationship should be the MIA. We expected to conduct a more comprehensive evaluation of the MIA combined with the IMPA to guide the range of lower incisor decompensation during presurgical orthodontics. This study aimed to systematically explore the MIA in severe skeletal class III patients.

## Materials and methods

### Subjects

This was a retrospective study. A total of 198 skeletal class III patients (99 men, 99 women) who underwent orthodontic treatment at Peking University School and Hospital of Stomatology were included. This retrospective study was approved by the Institutional Ethics Committee (Grant No. 2022- Beijing Municipal Science -26).

The inclusion criteria were as follows:(1) severe skeletal class III patients, ANB ≤ -4°at pretreatment (more than 2 standard deviations below the average value) and (2) availability of pretreatment lateral cephalometric radiographs that were of adequate quality.

According to the mandibular plane to cranial base (sella-nasion) angle (MP-SN), the patients were divided into three groups: the low-angle group with MP-SN < 27.3°(29 patients; 17 men, 12 women), the normal-angle group with 27.3° ≤ MP-SN ≤ 37.7°(104 patients; 55 men, 49 women), and the high-angle group with MP-SN > 37.7°(65 patients; 27 men, 38 women).

### Cephalometric analysis

**Fig. 1:**
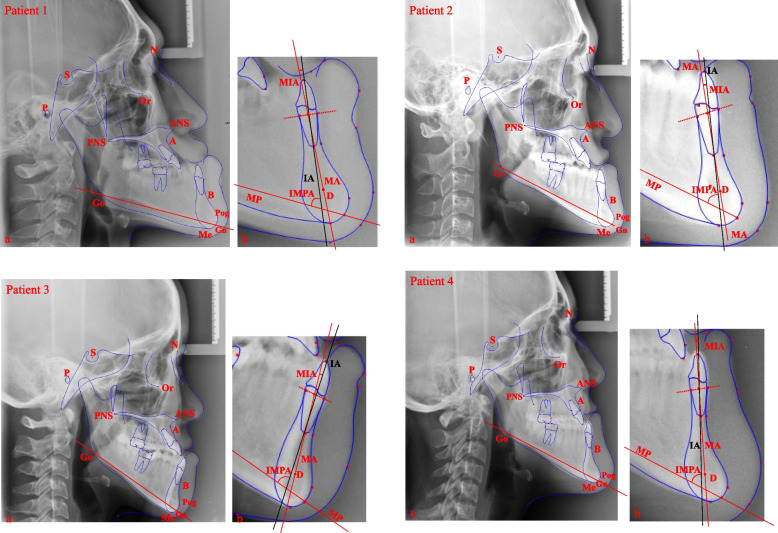
Examples of cephalometric digitization in severe skeletal class III patients before treatment. **a **Illustration of cephalometric landmarks and the mandibular plane (MP). **b **Illustration of the MIA and IMPA measurements. IA: the long axis of the lower central incisor; D: the centre point of the mandible symphysis; dotted line: the line passing through the labial and lingual lower alveolar edge of the lower central incisor; MA: the line from the midpoint of the line between the labial and lingual lower alveolar edge and point D; MIA: the angle between the IA and the MA; IMPA: the angle between the long axis of the lower central incisor (IA) and the MP

Before treatment, all lateral cephalograms were taken in the natural head position with the teeth in centric occlusion and lips relaxed as determined by Burstone [14]. To increase the reliability, all lateral cephalograms were obtained from the same cephalostat. After the cephalograms were obtained, two trained and calibrated investigators who were blinded to subject information used Dolphin Imaging® 10.0 software to locate the cephalometric landmarks two times each. For each subject, we measured 12 cephalometric parameters. The cephalometric landmarks and reference planes are shown and explained in Fig. 1, and the measured cephalometric parameters are presented in Table 1.

**Table 1:** Definitions of the cephalometric variables investigated in this study

Variable	Definition
SNA(°)	the sagittal relationship of the maxilla to the cranial base
SNB(°)	the sagittal relationship of the mandible to the cranial base
ANB(°)	the sagittal relationship between the maxilla and mandible relative to the cranial base
U1-NA(°)	the angle between the long axis of the upper central incisor and the NA line
SN-OP(°)	the angle between the cranial base and the occlusal plane
L1-NB(°)	the angle between the long axis of the lower central incisor and the NB line
U1-L1(°)	the angle between the long axis of the upper central incisor and the long axis of the lower central incisor
U1-SN(°)	the angle between the long axis of the upper central incisor and the cranial base
MP-SN(°)	the angle between the mandibular plane and the cranial base
FH-MP(°)	the angle between the mandibular plane and the Frankfort plane
MIA(°)	the angle between the MA and IA; MA:the long axis of the mandibular symphysis; IA:the long axis of the lower central incisor; when the IA is to the labial side of the MA, the MIA is a positive value
IMPA(°)	the angle between the long axis of the lower central incisor and the mandibular plane

**Fig. 2:**
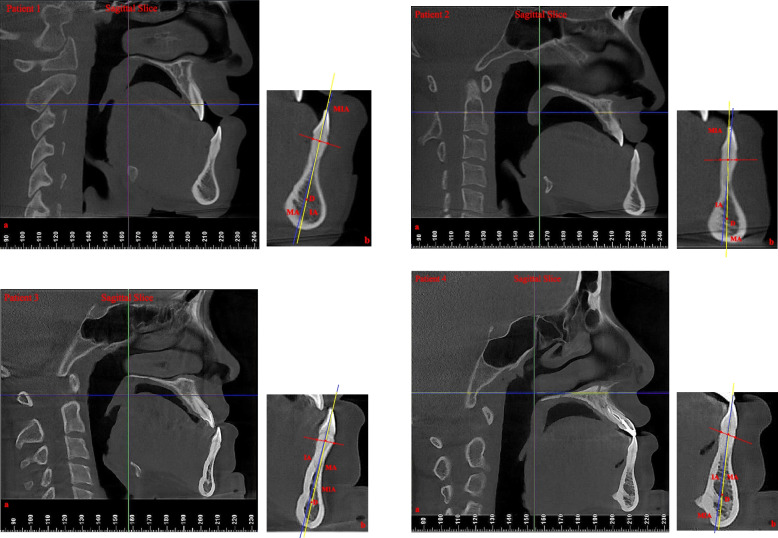
Examples and illustration of the CBCT measurement of the MIA in severe skeletal class III patients. IA:the long axis of the lower central incisor; D:the centre point of the mandible symphysis; dotted line: the line passing through the labial and lingual lower alveolar edge of the lower central incisor; MA: the line from the midpoint of the line between the labial and lingual lower alveolar edge and point D; MIA: the angle between the IA and the MA

CBCT scans were also obtained for each subject before treatment and were recorded using the same machine. We selected 11 samples randomly and measured their MP-SN, angle between the mandibular plane and the Frankfort plane (FH-MP) and MIA using Dolphin Imaging® 10.0 software in comparison with the cephalometric measurements to investigate the reliability of the cephalometric analysis. The MIA measurements are shown in Fig. 2.

#### Statistical analysis

All measurements were conducted by two trained investigators. Cephalometric and CBCT measurements were used for analysis. One-way analysis of variance (ANOVA) was applied to compare the cephalometric measurements of the MIA and IMPA in the three groups. The difference in the MIA and IMPA between sexes was analysed with an independent-samples t test. A paired-samples test was used to compare the differences between the cephalometric and CBCT measurements. All statistical analyses were performed with SPSS 26.0 (SPSS, IBM©, Armonk, NY, USA), and the *P* values were set at 0.05.

## Results

The means and standard deviations of the cephalometric measurements in the 3 groups (low, normal, and high angle) are shown in Table 2. Comparisons of the cephalometric measurements of the MIA and IMPA among the three groups and comparisons of the MIA and IMPA between sexes are shown in Table 3. CBCT measurements are shown in Table 4.

**Table 2:** Cephalometric measurements

Variable	Low-angle group(*n* = 29)		Normal-angle group(*n* = 104)		High-angle group(*n* = 65)	
	Mean	SD	Mean	SD	Mean	SD
SNA(°)	83.61	3.89	81.33	3.44	78.97	3.15
SNB(°)	90.49	3.77	87.80	4.01	84.04	3.20
ANB(°)	-6.87	2.14	-6.49	2.15	-5.07	1.36
U1-NA(°)	41.47	17.78	38.32	18.93	37.87	20.88
SN-OP(°)	6.15	6.90	12.88	4.18	19.19	4.51
L1-NB(°)	15.57	6.43	16.49	7.04	19.89	7.78
U1-L1(°)	132.48	10.29	135.88	15.68	131.70	11.53
U1-SN(°)	122.44	8.77	116.41	8.78	112.46	6.62
MP-SN(°)	24.08	3.47	33.07	3.06	41.98	3.70
FH-MP(°)	18.73	4.17	26.03	4.03	32.99	5.08
MIA(°)	3.70	5.26	3.52	5.61	2.33	5.97
IMPA(°)	80.78	7.04	75.51	7.67	73.85	7.92

**Table 3:** Comparison of the MIA and IMPA among the low-angle, normal-angle, and high-angle groups and comparison of the MIA and IMPA between sexes

	Low-angle group	Normal-angle group	High-angle group	ANOVA test *P* value	Male	Female	Independent-samples t test *P* value
	*n* = 29 (L)	*n* = 104 (N)	*n* = 65 (H)		*n* = 99(M)	*n* = 99(F)	
	Mean ± SD	Mean ± SD	Mean ± SD		Mean ± SD	Mean ± SD	
MIA(°)	3.70 ± 5.26	3.52 ± 5.61	2.33 ± 5.97	0.360	75.63 ± 8.73	75.84 ± 7.12	0.853
IMPA(°)	80.78 ± 7.04	75.51 ± 7.67	73.85 ± 7.92	0.001 L vs. N 0.000 L vs. H 0.170 N vs. H (LSD *P* value)	2.92 ± 5.83	3.39 ± 5.56	0.571

**Table 4:** Comparison of the cephalometric and CBCT measurements

Variable	Cephalometric measurement		CBCT measurement		Paired-samples t test *P* value
	Mean	SD	Mean	SD	
MP-SN(°)	33.81	3.93	32.86	3.43	0.816
FH-MP(°)	26.43	3.60	26.14	2.81	0.660
MIA(°)	5.01	4.36	5.92	3.93	0.142

The angular measurements SNA and SNB indicate the anteroposterior position of the maxilla and mandible relative to the cranial base, respectively. Compared with the range of normal values, approximately 64.1% showed protrusive mandibles, 18.7% showed a retrusive maxilla, and 8.1% showed a combination of protrusive mandible and retrusive maxilla in these severe skeletal class III subjects who needed combined orthodontic and orthognathic treatment. Two measurements of the inclination of maxillary incisors (U1-NA and U1-SN) showed significant proclination in these subjects. Two measurements of the inclination of mandibular incisors (L1-NB and IMPA) showed significant retroclination in these subjects. The mean of the interincisal angle was greater in these severe skeletal class III subjects than the normal range (Table 2).

The mean IMPA showed a significant difference among the three groups, while the differences in the mean MIA were not significant. In the 198 severe skeletal class III patients, the mean IMPA decreased with increasing MP-SN, which indicates that mandibular incisors were more lingually inclined for compensation. The low-angle group and normal-angle group showed mean MIA values of 3.70° and 3.52°, respectively, while the mean MIA (2.33°) was smaller in the high-angle group.

ANOVA showed no statistically significant difference in the pretreatment MIA among the low-angle, normal-angle and high-angle groups. However, there were statistically significant differences in the IMPA among the three groups (*P* < 0.001). Therefore, post hoc tests were performed to compare each vertical facial type with the others using the LSD-t method. The mean IMPA in the low-angle subjects (80.78 ± 7.04°) was significantly greater than that in the normal-angle (75.51 ± 7.67°, *P* = 0.001) and high-angle subjects (73.85 ± 7.92°, *P* < 0.001). Independent-samples t tests showed no statistically significant difference between sexes (Table 3).

The paired-samples t test showed that there were no statistically significant differences between the cephalometric and CBCT measurements of the MP-SN, FH-MP and MIA. The reliability of the cephalometric analysis was investigated (Table 4).

## Discussion

In the 198 severe skeletal class III patients included in this study, approximately 64.1% showed protrusive mandibles, 18.7% showed a retrusive maxilla, and 8.1% showed a combination of protrusive mandible and retrusive maxilla. Our data indicate that a protrusive mandible is the main cause of skeletal class III malocclusion in almost 72.2% of cases. From the cephalometric analysis, we can see that the maxillary incisors have a significant proclination and the mandibular incisors have a significant retroclination, which indicates that the maxillary incisors are labially inclined and the mandibular incisors are lingually inclined for compensation in these severe skeletal class III subjects.

For severe skeletal class III patients, orthodontic camouflage treatment cannot always achieve a good therapeutic effect, and orthodontists tend to choose combined orthodontic and orthognathic treatment. Previous studies have revealed that in skeletal class III malocclusion, the alveolar bone around incisors is originally or developmentally thinner than in normal occlusion, and there was still further absorption during presurgical treatment, particularly the alveolar bone around the mandibular anterior teeth [3, 4, 15–17]. Mandibular incisors, which are lingually inclined for compensation in skeletal class III patients, are more susceptible to recession of the labial gingiva and decreases in alveolar bone thickness (ABT) and height during presurgical orthodontic treatment [11, 18]. Preoperative orthodontic treatment aims to decompensate the maxillary and mandibular incisors to obtain normal and healthy tooth axial inclinations within their alveolar bone base. Because teeth must move through the alveolar bone house, orthodontists need to pay special attention to the morphology of the anterior alveolar bone and design a safe treatment plan to achieve a balance between the health of the alveolar bone and the outcome of orthognathic surgery. Therefore, an indicator is needed to evaluate the inclination of the mandibular incisors. Then, the necessary amount of incisor decompensation can be determined, and the already expressed compensation can be eliminated appropriately to facilitate surgical movements.

**Fig. 3:**
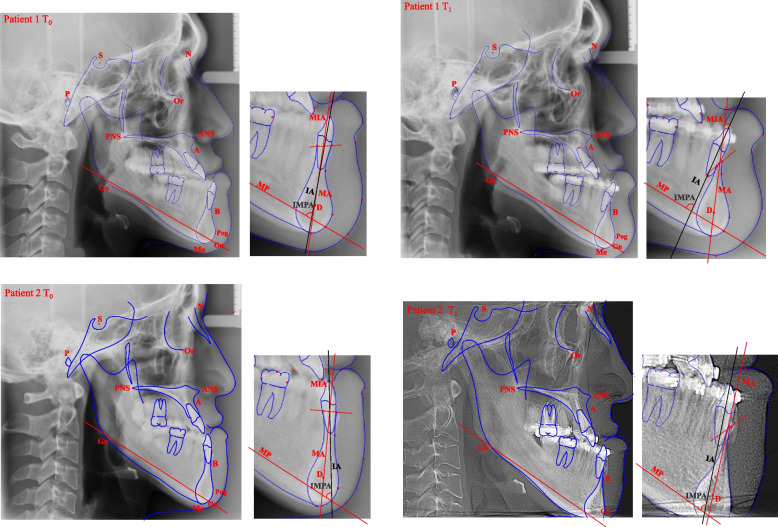
Examples of cephalometric digitization in severe skeletal class III patients before (T0) and after(T1) presurgical decomposition orthodontic treatment. The two patients’ lower central incisors exceeded the alveolar bone house after decomposition. IA:the long axis of the lower central incisor; D:the centre point of the mandible symphysis; dotted line: the line passing through the labial and lingual lower alveolar edge of the lower central incisor; MA: the line from the midpoint of the line between the labial and lingual lower alveolar edge and point D; MIA: the angle between the IA and the MA; IMPA: the angle between the IA and the MP

**Fig. 4:**
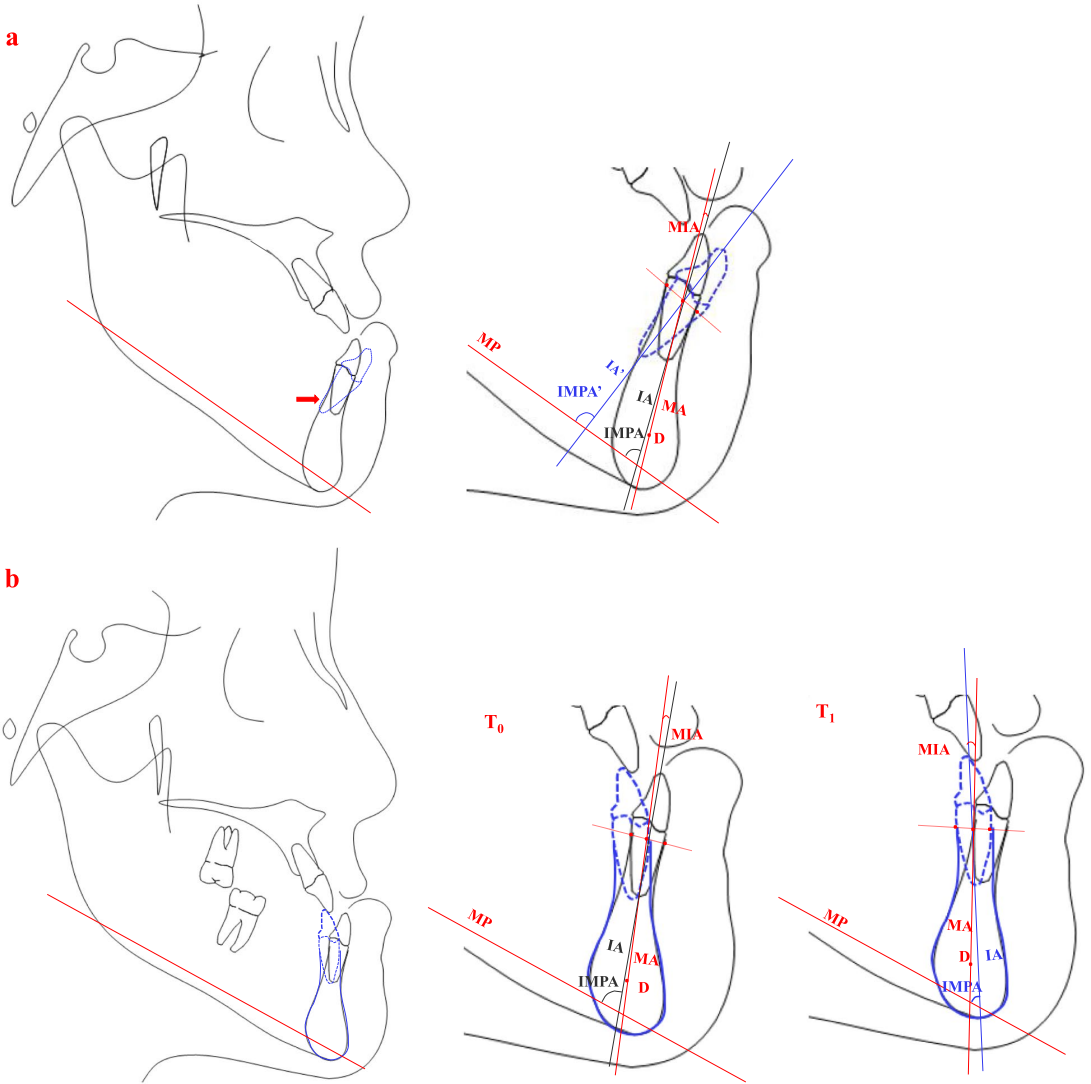
**a** Illustration of changes in alveolar bone and tooth movement of the lower central incisor after complete decomposition in presurgical orthodontic treatment. The lower central incisor indicated by the dotted line presents complete decompensation; red arrow indicates fenestration; IA and IA’:the long axis of the lower central incisor before and after decompensation; IMPA and IMPA’: the angle between the long axis of the lower central incisor and the MP before and after decompensation; MA:the long axis of the mandibular symphysis; MIA: the angle between the IA and the MA. **b **Illustration of physiological reconstruction of alveolar bone and tooth movement of the lower central incisor after orthodontic camouflage treatment by the force transmission technique. Fig.T_0_ and T_1_: the MIA and IMPA before and after treatment

The IMPA was traditionally used to evaluate the sagittal axial inclination of the mandibular central incisors. Tweed [19, 20] indicated that the IMPA was essential for facial aesthetics and tooth stability. He emphasized that the lower incisor should stand upright in the lower alveolar bone, believing that only in this way can the profile be perfect and the lower incisor be in a balanced position in the mandible. In this study, the IMPA of the untreated severe skeletal class III patients was generally significantly lower than the normal value to compensate for the negative overjet. With the increase in the MP-SN, the mean IMPA decreased significantly (Table 3). Studies have shown that the alveolar bone around incisors becomes thinner with the discrepant growth of jaws and the developmental compensation of teeth [4]. As shown in Figs. 3 and 4a, during preoperative treatment in severe skeletal class III cases, decompensation was performed completely based on the index IMPA to guarantee that the lower incisors standing upright in the mandible might cause the incisors to exceed the alveolar bone house, leading to more severe alveolar bone loss, fenestration, gingival recession and other unacceptable side effects [11, 21]. It is known that the combination of dental implants and alveolar bone is osseointegration and that mechanical force is transferred to the supporting bone. Studies have shown that the angle of force application and implant offset on the supporting bone significantly affect the stress on the supporting bone. Changes in the angle of force application result in greater stress on the supporting bone. The least stress in the supporting bone was found with vertical loading of no-offset implants [22, 23]. In Figs. 3 and 4a, after complete decompensation, the lower central incisor axis stayed away from the long axis of the mandibular symphysis. The transfer of occlusal force is unfavourable. However, in skeletal class III patients treated by the force transmission technique for camouflaging skeletal deformity, as shown in Fig. 4b, the consistency between the lower incisor axis and the long axis of the mandibular symphysis was maintained after treatment, although the lower incisor was more lingually inclined according to the IMPA. Light force induced physiological reconstruction of the alveolar bone, which is beneficial for the transfer of occlusal force.

In this study, the low-angle group and normal-angle group showed mean MIA values of 3.70° and 3.52°, respectively. The mean MIA (2.33°) was smaller in the high-angle group. There were statistically significant differences among the low-angle, normal-angle and high-angle groups (Table 3). We can conclude that under untreated physiological conditions, although the lower incisors were lingually inclined for compensation, the long axis of the mandibular incisors was highly consistent with the long axis of the mandibular symphysis, which was more obvious in the high-angle subjects. Compared with the angle between the inclination of the mandibular incisors and the mandibular plane, we should pay more attention to whether the mandibular incisors stand upright in the mandibular symphysis in skeletal class III malocclusion. From a physiological and functional point of view, the MIA better reflects the relationship between mandibular incisors and their alveolar bone house. During preoperative orthodontic treatment, we should not only be concerned with whether the lower incisor is standing upright in the mandible, but also pay special attention to the morphology of the anterior alveolar bone and maintain the lower incisor’s lingual inclination to some extent, which is more practical and safer.

Two-dimensional X-ray lateral cephalograms limit cephalometric analysis to linear and angular measurements between landmarks superimposed onto a single plane of space, often leading to distortion errors. Relatively speaking, investigators can visualize and measure the true 3-dimensional anatomy of patients from 3-dimensional CBCT scans, which avoids the intrinsic weaknesses of 2-dimensional imaging (distortion, superimposition, and investigators) [24, 25]. Studies have been performed to evaluate the accuracy and reliability of CBCT measurements. Leung et al. [25] reported that there is no significant difference between CBCT linear measurements and physical measurements, and Timock et al. [26] reported that CBCT can be used to quantitatively assess alveolar bone height and thickness with high precision and accuracy. Therefore, in addition to cephalometric analysis, we also randomly selected CBCT images of 11 subjects and measured their MP-SN, FH-MP and MIA in comparison with the cephalometric measurements to investigate the reliability of the cephalometric analysis. Paired-samples t tests indicated that there were no statistically significant differences between the cephalometric and CBCT measurements of the MP-SN, FH-MP and MIA. The reliability of the cephalometric analysis was investigated (Table 4).

The major limitation of the study was the sample size. The conclusions of this study are limited to 198 severe skeletal class III malocclusions, and we selected 11 samples randomly and assessed their CBCT image measurements for validation. Further studies should expand the sample size.

## Conclusion

In severe skeletal class III patients, the long axis of the mandibular incisors was highly consistent with the long axis of the mandibular symphysis, which was more obvious in the high-angle subjects.The MIA reflects the physiological inclination of the mandibular central incisors better than the IMPA.

## Data Availability

All data and their sources are clearly shown.

## Abbreviations

MIA: The angle between the long axis of the lower central incisor and the line from the midpoint of the line of the labial and lingual lower alveolar edge and the centre point of the mandible symphysis
IMPA: The angle between the long axis of the lower central incisor and the mandibular plane. The cephalometric variables are explained in Table 1
